# A New Tool for Public Health Opinion to Give Insight Into Telemedicine: Twitter Poll Analysis

**DOI:** 10.2196/13870

**Published:** 2019-05-28

**Authors:** Josep Vidal-Alaball, Luis Fernandez-Luque, Francesc X Marin-Gomez, Wasim Ahmed

**Affiliations:** 1 Health Promotion in Rural Areas Research Group Gerència Territorial de la Catalunya Central Institut Català de la Salut Sant Fruitós de Bages Spain; 2 Unitat de Suport a la Recerca de la Catalunya Central Fundació Institut Universitari per a la recerca a l'Atenció Primària de Salut Jordi Gol i Gurina Sant Fruitós de Bages Spain; 3 Qatar Computing Research Institute Hamad Bin Khalifa University Doha Qatar; 4 Newcastle Business School Northumbria University Newcastle upon Tyne United Kingdom

**Keywords:** telemedicine, Twitter messaging, health care surveys

## Abstract

**Background:**

Telemedicine draws on information technologies in order to enable the delivery of clinical health care from a distance. Twitter is a social networking platform that has 316 million monthly active users with 500 million tweets per day; its potential for real-time monitoring of public health has been well documented. There is a lack of empirical research that has critically examined the potential of Twitter polls for providing insight into public health. One of the benefits of utilizing Twitter polls is that it is possible to gain access to a large audience that can provide instant and real-time feedback. Moreover, Twitter polls are completely anonymized.

**Objective:**

The overall aim of this study was to develop and disseminate Twitter polls based on existing surveys to gain real-time feedback on public views and opinions toward telemedicine.

**Methods:**

Two Twitter polls were developed utilizing questions from previously used questionnaires to explore acceptance of telemedicine among Twitter users. The polls were placed on the Twitter timeline of one of the authors, which had more than 9300 followers, and the account followers were asked to answer the poll and retweet it to reach a larger audience.

**Results:**

In a population where telemedicine was expected to enjoy big support, a significant number of Twitter users responding to the poll felt that telemedicine was not as good as traditional care.

**Conclusions:**

Our results show the potential of Twitter polls for gaining insight into public health topics on a range of health issues not just limited to telemedicine. Our study also sheds light on how Twitter polls can be used to validate and test survey questions.

## Introduction

### Telemedicine

Telemedicine draws on information technologies in order to enable the delivery of clinical health care from a distance [1,2]. Telemedicine has been utilized around the world and a recent World Health Organization survey found that 38% of countries worldwide had some kind of telemedicine system and 30% had agencies that managed telemedicine services [1]. Telemedicine is particularly attractive in rural health areas as well as across long distances where it can be difficult to reach patients [2]; it has also been shown to increase the resolution of primary care teams, reducing referrals to face-to-face dermatology services [3]. There have been positive and unsuccessful implementations of telemedicine around the world [4]. This has likely led members of the public to have varying views and opinions toward the technology. It is important to gain an understanding of perceptions toward telemedicine before implementing an order to ensure it is received positively.

### Twitter for Public Health–Related Research

Twitter is a social networking platform that has 316 million monthly active users and its potential for real-time monitoring of public health has been well documented [5]. Anyone over the age of 13 with an Internet connection can register with Twitter. Upon registration, Twitter users can select a user handle, which begins with the “@” symbol (eg, @jmirpub). Once users are registered with the platform, it is possible to send short, 280-character text updates known as *tweets*; 500 million tweets are posted per day [5]. Twitter users can also follow other users and share their tweets, which is known as a *retweet*. One of the differences between Twitter and Facebook is that the majority of Twitter accounts are completely public. Twitter also has the concept of hashtags, expressed by placing the “#” symbol at the start of a word and allowing users to categorize a topic and form discussions around it. For instance, it is possible to search Twitter for the hashtag “#telemedicine,” which will display all tweets that have used the hashtag.

Twitter has been used previously as a platform to disseminate guidelines and perform polls by the European Association of Urology [5,6]. Twitter contains discussions related to a vast number of health topics, and a recent study noted that there were discussions on Twitter related to at least 379 different health conditions [7]. The study stated that the top-20 health communities, by number of tweets, included autism, diabetes, dementia, and AIDS. Moreover, a recently published systematic review examining Twitter as a tool for health research found that Twitter has been used for public health surveillance, recruitment, and intervention [8].

However, there is a lack of empirical research that has critically examined the potential of Twitter polls for providing insight into public health. Twitter is a platform with a demographic that is college educated and where users are likely to be aware of new forms of technology. This makes examining opinions toward telemedicine an interesting case.

One of the benefits of utilizing Twitter polls is that it is possible to gain access to a large audience that can provide instant and real-time feedback. Moreover, Twitter polls are completely anonymized and it is not possible to learn the identity of a user completing a poll, nor is it possible for one user to vote on more than one occasion. However, it must be noted that Twitter users could hold several accounts that could allow a single user to vote several times. Our results will be of interest to health authorities, policy makers, and academics interested in telehealth. They are also likely to be of interest to health authorities around the world seeking low-cost, real-time survey methods, as well as researchers interested in a critical examination of Twitter polls.

The overall aim of this study was to better understand opinions related to telemedicine on Twitter and to assess the potential of Twitter polls to validate and test survey questions.

## Methods

**Figure 1:**
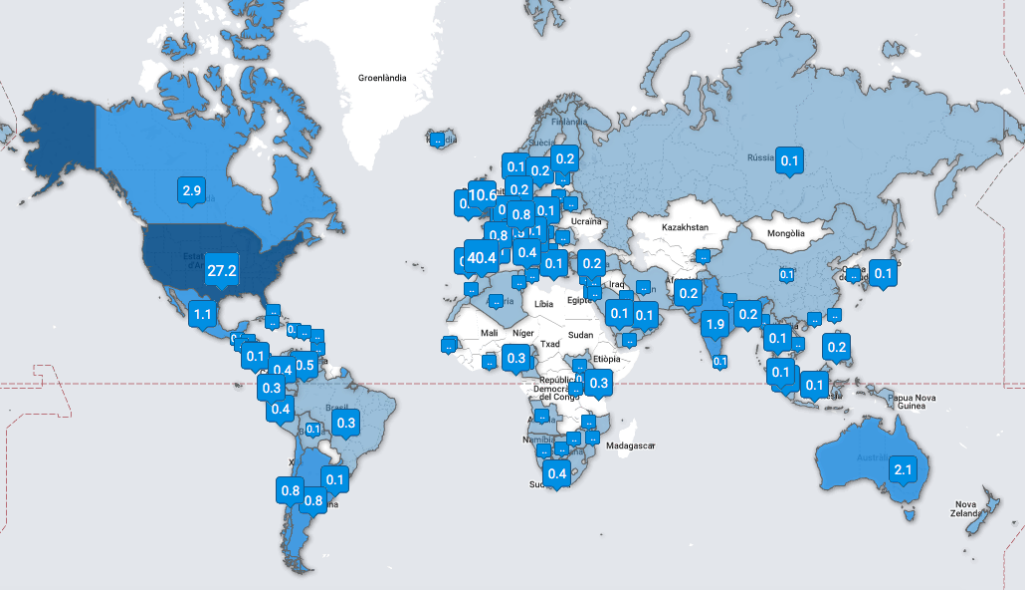
Map showing the percent distribution of Twitter followers by country.

In this study, we devised two Twitter polls using questions from previous questionnaires to explore acceptance of telemedicine among Twitter users. We distributed the polls on the Twitter timeline belonging to one of the authors and asked the followers of the account to answer the poll and retweet it to reach a larger audience. The Twitter handle that we used for this study, @jvalaball, has more than 9300 followers. The followers of the Twitter account that was utilized had a worldwide audience that mainly derived from Spain (40%), the United States (27%), and the United Kingdom (11%). Figure 1 displays the percent distribution of Twitter followers by country; the map was generated using the Web application TweepsMap [9]. Ethical approval was not required because Twitter polls are completely anonymized.

## Results

Our first Twitter poll was distributed in May 2016 and was *pinned* to the top of the Twitter timeline used in the project for 7 days. By pinning a tweet, it will permanently be placed at the top of a Twitter user’s account, such that any new visitor will see the tweet appear at the top of a user’s timeline.

**Figure 2:**
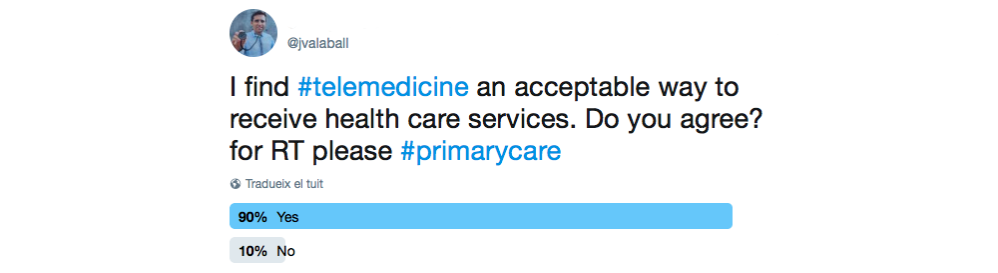
First Twitter poll (screenshot). RT: retweet.

We used a question from the telemedicine satisfaction questionnaire, a validated questionnaire developed by Yip et al in 2002 [10]. The question posted was as follows: “I find telemedicine an acceptable way to receive health care services. Do you agree?” For the responses, only two answers were allowed, which were *Yes* or *No*. Figure 2 displays how the tweet was constructed as well as the responses submitted by Twitter users.

The poll was retweeted 51 times and had 6698 impressions (ie, number of views). It received a total of 108 votes, 89.8% (97/108) of which were positive and 10.2% (11/108) negative.

**Figure 3:**
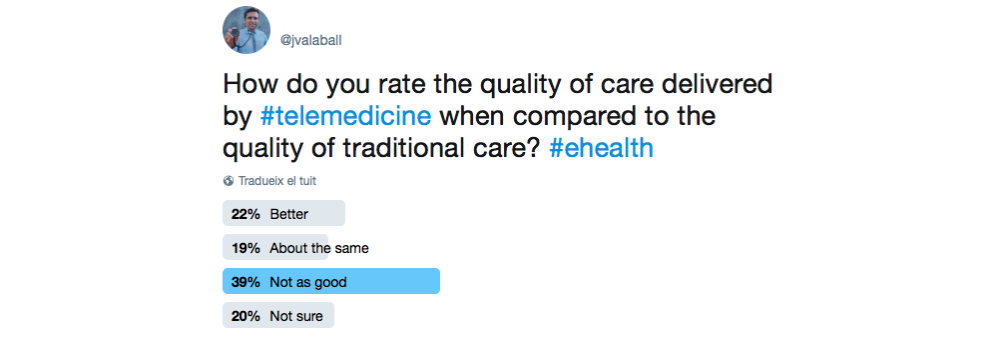
Second Twitter poll (screenshot).

The second poll was posted during November 2017, and it was also pinned to the top of the timeline for 7 days. For this Twitter poll, we used a question from the physician questionnaire in the European Union project Health Optimum [11]. The question posted was as follows: “How do you rate the quality of care delivered by telemedicine when compared to the quality of traditional care?” Four answers were allowed: *Better*, *About the same*, *Not as good*, and *Not sure*. The poll was retweeted 49 times and had 4364 impressions. Figure 3 provides insight into how the tweet was constructed as well as the responses submitted by Twitter users.

Overall, the poll received a total of 113 votes. A total of 38.9% (44/113) of the respondents stated that they rated the quality of care delivered by telemedicine as *Not as good* as traditional care, 18.6% (21/113) found the quality of care to be *About the same*, 22.1% (25/113) rated the quality of care as *Better*, and 20.4% (23/113) responded that they were *Not sure* about the level of care.

## Discussion

Our study provided an overview of a novel experiment where targeted Twitter polls were used to assess acceptance of telemedicine among Twitter users. We argue that this tool could be used to quickly perform surveys to assess the opinion of users regarding acceptance of telemedicine in order to obtain rapid feedback of new questionnaires before validating them. An advantage of using Twitter polls is that many can be created and disseminated in very little time as opposed to traditional questionnaires and surveys, which can become resource heavy. One of the key benefits of utilizing social media platforms such as Twitter is the very low cost when compared to traditional survey-based methods. In certain departments with low budgets, Twitter could be used as a tool to gain initial public opinion feedback before a survey could be devised. The first poll showed an overwhelming support toward telemedicine as an acceptable way to receive health care services. In the second poll, which asked Twitter users to rate the quality of care delivered by telemedicine when compared to the quality of traditional care, the majority of users found that telemedicine was not as good as traditional care. This highlights how the design of a question can potentially influence the results of a survey. Our method could be used to conduct testing on survey questions and to compare the answers to ensure they are consistent. Although the two Twitter polls received a very high number of impressions—6698 and 4364, respectively—the response rates were comparatively low. This could be due to the fact that the questions were not attractive enough to the audience for them to feel compelled to reply. It must be noted that one of the limitations of using Twitter for gauging public opinion through the use of Twitter polls is that its users are not representative of the general population in terms of demographics [12]. A further limitation of Twitter polls is that users with multiple accounts can vote on more than one occasion. Moreover, users with malicious intentions could attempt to manipulate the poll by using multiple accounts to repeatedly vote. However, a growing body of literature is noting the potential of social media data for providing unfiltered public opinion [13-15]. This is because one of the potential strengths of using social media data has been the ability to avoid the risk of interview bias [13]. Furthermore, due to the ability of social media to set agendas in mainstream media [16,17], it can be argued that it has become important to study content and public opinion held by social media users.

Our study has demonstrated the potential of Twitter polls for gaining insight into public health topics such as telemedicine and for shedding light on how Twitter polls can be used to validate and test survey questions. Twitter polls could be utilized by health authorities to gain early real-time feedback on public views and opinions on a range of health issues not just limited to telemedicine. Key strengths are the speed at which Twitter polls can be formulated as well as their low cost.

## Abbreviations

RT: retweet
